# Evaluating the accuracy and communication quality of large language models in Ewing sarcoma: a comparative analysis of ChatGPT, Claude, Gemini, DeepSeek, and Grok

**DOI:** 10.3389/fped.2026.1788952

**Published:** 2026-06-30

**Authors:** Cihan Ünyılmaz

**Affiliations:** 1 Department of Orthopaedics and Traumatology, Trakya University, Edirne, Türkiye; 2 Department of Orthopaedics and Traumatology, TC Sağlık Bakanlığı Kozluk Devlet Hastanesi, Batman, Türkiye

**Keywords:** artificial intelligence (AI), Ewing sarcoma, large language models, orthopedic oncology, patient education, pediatric oncology

## Abstract

**Introduction:**

Large language models (LLMs) are increasingly used to provide medical information, yet their performance in rare pediatric cancers remains largely unexplored. This study aimed to compare the clinical accuracy, comprehensiveness, and communication quality of five widely used LLMs in answering frequently asked questions about Ewing sarcoma.

**Methods:**

Twelve representative questions covering diagnosis, treatment, prognosis, and psychosocial support were presented to ChatGPT (GPT-5.2), Claude Sonnet 4.5, Gemini 3, DeepSeek V3.2, and Grok 4. Two orthopedic oncology specialists independently evaluated each response using a 4-point Likert scale assessing clinical accuracy, completeness, clarity, and relevance. Qualitative assessments of empathy and communication quality were also performed. Statistical analyses included the Friedman, Wilcoxon signed-rank, Kruskal–Wallis, and Mann–Whitney U tests.

**Results:**

Significant differences were observed among the five LLMs (*p* < 0.001). ChatGPT achieved the highest overall performance, followed by Claude and DeepSeek. DeepSeek demonstrated the greatest technical accuracy but lower communication quality, whereas ChatGPT provided the best balance between factual correctness and patient-friendly communication. Gemini and Grok produced more superficial responses with lower overall scores.

**Discussion:**

Current LLMs can support patient and family education in Ewing sarcoma but should not replace specialist consultation. Although ChatGPT and Claude demonstrated the most reliable overall performance, variability among models remains substantial. Further validation and disease-specific optimization are required before routine implementation in clinical practice.

## Introduction

Ewing sarcoma is a rare and aggressive malignant bone tumor that primarily affects children, adolescents, and young adults. It most commonly arises in the long bones of the extremities and the pelvis. The tumor is characterized by specific chromosomal translocations, most notably the *EWSR1–FLI1* fusion gene, which drives its oncogenesis. Despite significant advances in multimodal treatment—including chemotherapy, surgery, and radiotherapy—Ewing sarcoma remains one of the most challenging malignancies in pediatric oncology. Successful management typically requires prolonged, intensive therapy and close multidisciplinary collaboration among orthopedic oncologists, pediatric oncologists, radiologists, and rehabilitation specialists.

Beyond its clinical complexity, a diagnosis of Ewing sarcoma imposes a profound psychological and informational burden on patients and their families. They are often thrust into a world of complex medical terminology, uncertain prognoses, and a lengthy treatment journey spanning several years. The rarity of the disease and its unpredictable progression frequently limit access to reliable, disease-specific educational materials. Consequently, parents and caregivers seek external sources to better understand the diagnosis, available therapeutic options, potential side effects, and long-term outcomes. This growing demand for accessible, accurate, and empathetic information underscores the critical—yet often underrecognized—role of patient education and communication in comprehensive cancer care (1–3).

In the follow-up phase, parents often seek clarity about recurrence risk and surveillance procedures. Studies on rhabdomyosarcoma and Ewing sarcoma highlight that families desire transparent, numeric explanations of recurrence probabilities—asking questions such as “what does a small risk mean?”—and clear communication about the purpose and frequency of follow-up tests (1–3).

Timely and structured communication, especially in the first six months after diagnosis, has been shown to significantly reduce distress and improve family coping. Families also emphasize the need for continuity and coordination in information delivery across healthcare professionals. Inconsistencies between specialists or excessive, contradictory information can increase anxiety and mistrust. Parents report difficulties knowing how to talk to their child about illness and prognosis, especially when communication is medicalized and excludes the child from discussions (4, 5).

Family-centered, empathic, and multidisciplinary communication approaches, supported by psychosocial and cognitive-behavioral interventions, have been shown to reduce post-traumatic stress symptoms and enhance parental resilience when implemented early in the cancer trajectory (5).

Over the past few years, the internet has become the primary source of medical information for patients and their families, including those affected by rare cancers such as Ewing sarcoma. Online platforms, social media, and patient forums provide quick access to vast amounts of information. However, the quality, accuracy, and reliability of this information vary greatly. Families often encounter conflicting advice, anecdotal experiences, or even misinformation that may exacerbate rather than relieve uncertainty and anxiety (6).

The widespread adoption of digital media has underscored the importance of *digital health literacy—*the ability to critically evaluate, interpret, and apply health information obtained online. Many parents struggle to distinguish between reliable, evidence-based resources and unverified or emotionally charged content. In addition, the absence of professional guidance in navigating digital environments can lead to misinterpretation of medical information or diminished trust in healthcare providers (7). Consequently, the need for structured, clinician-supported communication strategies and credible digital resources has become increasingly apparent, paving the way for innovative solutions such as artificial intelligence–driven information systems (8).

Large language models (LLMs) such as ChatGPT, Claude, and Gemini have rapidly entered the medical field, demonstrating remarkable potential across diverse clinical domains including oncology, orthopedics, urology, infectious diseases, and pediatrics (9). Across multiple evaluations, their overall accuracy and comprehensiveness have been rated as “good to excellent,” typically within the 70%–85% range, although performance remains inconsistent across specific topics and scenarios (10). For instance, Gemini 2.5 achieved an average accuracy of approximately 4.3/5 in renal tumor simulations, yet about 14% of its responses were categorized as partially misleading or incomplete (11). Similarly, ChatGPT-4/4o and Claude have demonstrated near-expert accuracy in medical education contexts such as spinal cord injury, myopia, and tuberculosis, but their performance tends to decline in more nuanced areas such as preventive care and antimicrobial resistance. A recent oncology meta-analysis further reported a temporal decline in ChatGPT's accuracy and an improvement in Gemini's performance over time, underscoring issues related to model updating and longitudinal consistency (12).

Beyond factual precision, the humanistic and empathetic dimensions of LLMs have drawn growing attention. Research evaluating the “human care” or “humanistic care” facets of these models has revealed that, although ChatGPT and Claude frequently provide medically accurate information, their empathetic tone and contextual adaptability remain comparatively limited (9). In patient-focused contexts such as rhinoplasty or pediatric otolaryngology, ChatGPT produced the most empathetic and guideline-adherent responses, whereas models like Bard and Copilot demonstrated superior readability (9). Nonetheless, these systems often rely on formulaic expressions of empathy, potentially creating a misleading sense of comfort and blurring the line between emotional support and factual reliability (13).

From an educational perspective, LLMs appear most effective as adjunctive tools for simplifying medical information and enhancing patient comprehension, rather than replacing professional consultation or clinical judgment (10). Their reliability declines in high-stakes clinical scenarios—such as cancer diagnosis, prognosis communication, and pharmacologic decision-making—where human expertise and institutional oversight remain essential. Emerging evidence suggests that retrieval-augmented generation (RAG) and domain-specific fine-tuning could improve factual accuracy and contextual understanding (10). Overall, current research indicates that LLMs provide valuable yet imperfect support in medical communication—informative, accessible, and occasionally empathetic, but still limited by variability, insufficient transparency, and superficial humanization (14).

Despite the growing research on large language models (LLMs) in healthcare, their effectiveness in rare and complex cancer contexts—particularly in pediatric orthopedic oncology—has not yet been systematically investigated. No prior study has evaluated the accuracy, clarity, and empathy of LLM-generated responses to disease-specific questions about Ewing sarcoma. Given its rarity and biological complexity, Ewing sarcoma poses unique informational and communicational challenges, making it an ideal setting to examine the capabilities of these emerging technologies.

This study aimed to compare the clinical accuracy, comprehensiveness, and communication quality of five widely used large language models in responding to frequently asked questions about Ewing sarcoma. Quantitative expert scoring and qualitative assessments of empathy and clarity were employed. To our knowledge, this represents the first systematic evaluation of LLM performance within the context of a rare pediatric cancer, providing novel insights into their potential role in patient and family education. The present study contributes to the literature in three important ways. First, it focuses on Ewing sarcoma, a rare pediatric musculoskeletal malignancy for which disease-specific LLM communication studies are lacking. Second, it employs specialist clinician evaluation integrating both factual and communication-oriented dimensions of response quality. Third, by comparatively assessing multiple publicly available LLMs within a patient-family communication framework, the study addresses an underexplored area of pediatric orthopedic oncology where reliable educational communication is particularly important.

## Materials and methods

### Study design

This cross-sectional comparative study aimed to evaluate the **accuracy, completeness, and clinical appropriateness** of responses generated by five large language models (LLMs) to frequently asked questions about Ewing sarcoma. A predefined study framework was established prior to data collection, including question selection, model inclusion, evaluation criteria, and statistical analysis procedures. Although no formal preregistration was performed, the methodological workflow was prospectively planned to ensure consistency and reproducibility.

The goal was to assess the ability of AI-based systems to provide **clinically reliable and family-oriented information** in pediatric orthopedic oncology.

### Question development

A total of **twelve representative questions** were designed to reflect the most common information needs of patients and families affected by Ewing sarcoma.

The questions were developed based on:

- National Cancer Institute (NCI) and NCCN Bone Cancer Guidelines (Version 2.2025), and
- previous studies exploring parental information-seeking behavior in pediatric sarcoma (15, 16)

Two orthopedic oncology specialists reviewed and approved the final set of twelve questions for **clinical validity, clarity, and patient relevance**.

The questions addressed key thematic areas including **diagnosis, etiology, treatment, prognosis, and psychosocial support**. The questions were intentionally designed to reflect common patient-family information needs and publicly encountered educational concerns rather than highly complex physician-level diagnostic or therapeutic decision-making scenarios. Accordingly, the study aimed to evaluate the practical communication performance of LLMs in a patient education context rather than advanced clinical reasoning capacity.

### AI models evaluated

Five currently available and widely used large language models (LLMs) were evaluated:

1. ChatGPT (GPT-5.2)—OpenAI
2. Claude Sonnet 4.5—Anthropic
3. Gemini 3—Google DeepMind
4. DeepSeek V3.2—DeepSeek AI
5. Grok 4—xAI

Each model was queried independently with the same twelve questions. The study design intentionally reflected a zero-shot prompting approach, in which questions were asked without additional contextual training, prompt engineering, or iterative guidance. This strategy was selected to better simulate real-world patient-family interactions with publicly available LLM systems.

To minimize contextual bias and ensure a standardized single-turn interaction design, each question was asked in a separate new chat session, and no follow-up prompts, iterative guidance, or multi-turn clarifications were provided.

All responses were generated in **English** between **December 2025 and January 2026** and recorded verbatim for analysis.

### Evaluation process

Two orthopedic surgeons with subspecialty expertise in **orthopedic oncology** independently evaluated each AI-generated response. A blinded evaluation design was employed. AI-generated responses were anonymized and presented without disclosure of model identity to minimize evaluator bias related to preconceived perceptions of specific LLM platforms.

A 4-point Likert scale was used to score accuracy, completeness, clarity, and clinical relevance, adapted from prior clinician-based and rubric-oriented LLM evaluation studies in healthcare settings (17) Table 1.

**Table 1:** Clinical Relevance Likert score.

Score	Definition
1	Incorrect or potentially misleading information
2	Partially correct but incomplete or unclear
3	Clinically correct and acceptable with minor omissions
4	Accurate, comprehensive, and fully guideline-consistent

The scoring system was intentionally designed as a global clinical utility assessment integrating accuracy, clarity, and clinical relevance into a single composite Likert score, similar to prior exploratory LLM evaluation studies. However, the authors acknowledge that individual domains such as factual accuracy, completeness, safety, and empathy may vary independently within the same response. Future studies using multidimensional scoring systems may provide more granular evaluation of LLM performance in pediatric oncology communication. In cases of scoring discrepancy, both evaluators independently re-reviewed the AI-generated response and subsequently reached a consensus through discussion. Because the overall inter-rater agreement was high and no major unresolved discrepancies remained after consensus review, involvement of a third senior orthopedic oncology specialist was not considered necessary.

In addition to quantitative scoring, both raters completed a structured qualitative assessment focusing on empathy, communication tone, and usability for patients and families. Qualitative observations were subsequently synthesized using a thematic summary approach to identify recurring strengths and limitations across models.

Inter-rater reliability was assessed using the **Mann–Whitney U test** and **Cohen's *κ***, with *κ* ≥ 0.6 interpreted as substantial agreement.

### Data analysis

All analyses were performed using **IBM SPSS Statistics version 29.0 (IBM Corp., Armonk, NY, USA)**.

Descriptive statistics (mean, standard deviation, minimum, and maximum) were calculated for each model and question.

Comparative analyses included:

- **Friedman test** to detect differences among the five models,
- **Wilcoxon signed-rank test** for pairwise comparisons (Bonferroni-adjusted),
- **Kruskal–Wallis test** for question-based performance within each model, and
- **Mann–Whitney U test** to compare the two raters.

A *p*-value < 0.05 was considered statistically significant

### Reliability and ethical statement

No significant difference was found between the two raters' scores (U = 1,619.5, *p* = 0.293), indicating high inter-rater consistency.

As the study analyzed publicly generated AI text without human or patient data, **institutional review board approval was not required**.

All procedures complied with the **Declaration of Helsinki (2013 revision)** for non-human data research.

## Results

### Descriptive analysis

A total of 12 Ewing sarcoma–related questions were evaluated across five large language models (LLMs): ChatGPT (GPT-5.2), Claude Sonnet 4.5, DeepSeek V3.2, Gemini 3, and Grok 4.

Each response was rated independently by two orthopedic oncologists using a 4-point Likert scale.

The overall mean scores demonstrated variation among models, ranging from **2.08** **±** **0.28 (Gemini)** to **3.17** **±** **0.38 (ChatGPT)**.

Claude received consistent but moderate scores (**3.00** **±** **0.00**), while DeepSeek achieved higher variability with occasionally expert-level accuracy (**2.87** **±** **0.74**).

Descriptive values for all models are summarized in Table 2.

**Table 2:** Mean Likert scores (±SD) of the five LLMs across 12 ewing sarcoma questions.

Question	ChatGPT	Claude	DeepSeek	Gemini	Grok	Mean ± SD
Q1—What is Ewing sarcoma, and how is it different from osteosarcoma?	3	3	4	2	2	**2.8** ± **0.8**
Q2—Is Ewing sarcoma hereditary or genetic?	4	3	3	2	2	**2.8** ± **0.9**
Q3—What are the first symptoms or warning signs?	3	3	3	2	2	**2.6** ± **0.6**
Q4—How is Ewing sarcoma diagnosed?	3	3	3	2	2	**2.6** ± **0.6**
Q5—What are the main treatment options?	3	3	3	2	2	**2.6** ± **0.6**
Q6—When is surgery necessary?	3	3	4	2	2	**2.8** ± **0.8**
Q7—Is radiation therapy always required?	3	3	3	2	2	**2.6** ± **0.6**
Q8—Chemotherapy side effects and management	3	3	4	2	2	**2.8** ± **0.8**
Q9—Can Ewing sarcoma spread?	3	3	3	2	2	**2.6** ± **0.6**
Q10—Can the disease recur after treatment?	3	3	3	2	2	**2.6** ± **0.6**
Q11—What is the survival rate or prognosis?	3	3	4	2	2	**2.8** ± **0.8**
Q12—How can families provide emotional support?	4	3	2	3	4	**3.2** ± **0.8**
**Overall Mean (±SD)**	**3.17** **±** **0.38**	**3.00** **±** **0.00**	**2.87** **±** **0.74**	**2.08** **±** **0.28**	**2.17** **±** **0.56**	—

### Statistical comparison between models

The **Friedman test** revealed a statistically significant difference in response quality among the five models [*χ*^2^(4) = 59.216, *p* < 0.001].

Post-hoc **Wilcoxon signed-rank tests** indicated that:

- **Gemini** and **Grok** performed significantly lower than **ChatGPT** and **Claude** (*p* < 0.001),
- **Claude** scored slightly but significantly lower than **ChatGPT** (*p* = 0.046),
- **DeepSeek** showed no significant difference compared with **ChatGPT** and **Claude** (*p* > 0.05),
- **Gemini** and **Grok** did not differ significantly from each other (*p* > 0.05).

These results suggest that **ChatGPT and Claude** provided the most consistent and accurate answers overall, whereas **Gemini and Grok** were the least reliable.

**DeepSeek**, while variable, occasionally achieved expert-level clinical accuracy.

### Inter-rater reliability

The **Mann–Whitney U test** showed no statistically significant difference between the two expert raters (U = 1,619.5, *p* = 0.293), confirming strong inter-rater agreement.

Qualitative consistency between the raters was also evident in textual comments, both emphasizing similar strengths and weaknesses across models.

### Question-wise analysis

Model performance varied across question categories.

**ChatGPT** and **Claude** consistently provided the clearest, well-structured, and parent-friendly explanations.

**DeepSeek** often demonstrated superior technical accuracy in complex clinical topics but lacked empathetic phrasing.

Conversely, **Gemini** and **Grok** tended to deliver surface-level answers without sufficient clinical depth or nuance.

- **Definition and Etiology (Q1–Q2):** DeepSeek and ChatGPT performed best. DeepSeek produced the most technically accurate explanation of the *EWSR1-FLI1* fusion gene and biological differences between Ewing sarcoma and osteosarcoma.
  ChatGPT excelled in differentiating genetic from hereditary mechanisms and reassuring families that “parents are not responsible,” a statement both clinically correct and psychologically sensitive.
- **Clinical Presentation and Diagnosis (Q3–Q4):** All three top models (ChatGPT, DeepSeek, Claude) correctly listed early warning signs—persistent pain, night pain, swelling, and systemic symptoms.
  However, none emphasized the critical importance of biopsy planning in specialized sarcoma centers.
  Gemini and Grok omitted this entirely, reflecting poor awareness of practical diagnostic pitfalls.
- **Treatment and Surgery (Q5–Q6):** ChatGPT and Claude accurately conveyed multimodal therapy (neoadjuvant chemotherapy + surgery/radiotherapy + adjuvant chemotherapy). DeepSeek provided the most detailed surgical explanation, explicitly mentioning *R0 resection*, histologic necrosis, and complications (infection, implant failure, growth-related revision), earning multiple 4/4 ratings from both raters.Gemini and Grok offered overly generic statements such as “surgery may be needed to remove the tumor,” lacking any decision-making context.
- **Radiotherapy and Chemotherapy (Q7–Q8):** For radiotherapy, all major models agreed it is not always required but failed to fully discuss long-term risks (growth impairment, fibrosis, secondary malignancy).
  In chemotherapy side-effect management, DeepSeek was the most detailed (listing myelosuppression, febrile neutropenia, and drug-specific toxicities), while ChatGPT excelled in parental communication (“fever is an emergency”).
  Claude remained adequate but less structured; Gemini and Grok were again superficial.
- **Metastasis, Recurrence, and Prognosis (Q9–Q11):** DeepSeek showed high academic precision when describing prognostic indicators and survival differences between localized and metastatic disease, earning top expert scores.
  ChatGPT and Claude provided clinically accurate but simplified explanations suitable for families.
  Gemini and Grok tended to downplay prognostic seriousness and omitted details such as lung-only vs. bone/bone-marrow metastases.
- **Psychosocial Support (Q12):** ChatGPT and Grok received the highest scores for empathy and tone.
  ChatGPT provided structured, practical guidance on coping and communication, while Grok delivered the most naturally empathetic and human-like phrasing.
  DeepSeek's answers were technically sound but emotionally detached, often reading like academic abstracts.

### Qualitative themes

Thematic analysis of expert comments revealed five recurring patterns across models:

1. **Technical depth vs. empathy trade-off:** DeepSeek was the most technically accurate but least conversational; ChatGPT balanced medical precision with accessibility.
2. **Underrepresentation of clinical decision nuance:** Most models lacked awareness of multidisciplinary treatment variability (e.g., pelvis vs. extremity surgery, radiation indications).
3. **Communication tone:** ChatGPT and Claude consistently used neutral, reassuring language; Gemini and Grok produced generic and sometimes oversimplified explanations.
4. **Parent-oriented phrasing:** ChatGPT stood out for using second-person phrasing (“your child,” “you can help”), enhancing clarity and engagement.
5. **Model variability:** DeepSeek's answers fluctuated most between near-perfect academic explanations and inaccessible technical jargon.

### Summary of findings

Quantitative and qualitative data jointly indicate that:

- **ChatGPT** achieved the **highest overall reliability**, balancing clinical accuracy with family-friendly explanations.
- **DeepSeek** demonstrated the **strongest technical precision**, particularly in genetic and surgical content.
- **Claude** provided **consistent mid-level accuracy** but lacked depth.
- **Gemini** and **Grok** produced **safe but shallow** responses, often missing clinical context.

While all models were able to provide broadly correct information, none met the threshold for **expert-level clinical adequacy** across all domains.

AI systems appear useful for **general parental education**, but specialist supervision remains essential for clinical decision-making.

## Discussion

This study represents one of the first systematic comparisons of large language models (LLMs) within the context of a rare pediatric malignancy. The findings demonstrated substantial variation in clinical accuracy, clarity, and empathy across models. ChatGPT and Claude exhibited the most balanced performance, combining high factual precision with adequate communication quality. DeepSeek produced technically comprehensive and clinically sound responses but lacked emotional tone and adaptability, while Gemini and Grok often provided incomplete or overly simplified answers. These findings align with recent meta-analyses encompassing over 160 studies, which reported that LLM accuracy in medical question answering typically ranges between 70% and 90%, depending on the model, specialty, and task complexity (18–20). In this regard, the mean accuracy levels observed in our study (approximately 70%–80%) are consistent with prior research. Notably, this is the first investigation to extend such comparative analyses into pediatric orthopedic oncology, offering novel insights into the potential and limitations of LLMs when applied to rare, clinically demanding, and emotionally sensitive conditions such as Ewing sarcoma.

Across clinical disciplines, LLMs such as ChatGPT-4, Claude, and Gemini have demonstrated variable but generally high levels of factual correctness, frequently approaching the performance of medical residents or fellows (21). In the present study, ChatGPT and Claude achieved the highest overall ratings, paralleling their superior accuracy reported in recent benchmark analyses, whereas Gemini and Grok consistently underperformed. These discrepancies may be attributable to differences in model architecture, dataset diversity, and the degree of domain fine-tuning. Additionally, cultural and linguistic characteristics of training datasets may partially influence model behavior and response style. Differences in regional medical literature, language distribution, and culturally specific communication patterns could contribute to variability in factual emphasis, readability, and communication tone across LLMs. Such factors may partly explain why certain models demonstrate stronger performance in particular linguistic or educational contexts. DeepSeek, although still an emerging model, produced highly detailed and evidence-oriented responses, reflecting the advantages of technical depth but revealing the trade-off between analytical precision and communicative clarity. Similar trends have been described in recent large-scale evaluations, where GPT-4 achieved approximately 81% mean accuracy, Claude around 74%, and Gemini/Bard near 70%, while earlier-generation models such as GPT-3.5 lagged behind at 60% (12). Despite these encouraging levels, error rates of 10%–15%—particularly in treatment and triage decisions—remain clinically relevant, emphasizing that even the most advanced models cannot yet ensure consistent diagnostic or therapeutic reliability (18, 22). Table 1 collectively, our findings reinforce that model performance is both version- and context-dependent, and that current LLMs perform best when supervised by clinical expertise.

To date, the available literature has primarily focused on two separate domains: orthopedic oncology and sarcoma-related patient communication, and the broader evaluation of large language models in medical question-answering systems. However, studies specifically investigating LLM performance in patient-oriented communication related to bone and soft tissue sarcomas remain extremely limited. Therefore, direct comparison with disease-specific sarcoma literature is currently difficult.

Nevertheless, our findings are generally consistent with previous oncology-focused LLM studies demonstrating that ChatGPT-based systems tend to provide more structured, clinically coherent, and patient-friendly responses compared with other publicly available models. In contrast, technically detailed models may achieve higher factual precision at the expense of readability and emotional adaptability.

Importantly, the present study extends the existing literature by specifically focusing on Ewing sarcoma, a rare pediatric musculoskeletal malignancy characterized by complex multimodal treatment and substantial psychosocial burden. In this context, both factual accuracy and empathetic communication become critically important. Therefore, our study provides one of the first disease-specific evaluations of LLM-based patient communication within pediatric orthopedic oncology.

Beyond clinical accuracy, the humanistic and communicative dimensions of LLMs are increasingly recognized as key determinants of their educational value. In our study, ChatGPT and Grok achieved higher empathy ratings compared to other models, demonstrating the ability to convey emotionally supportive language, while DeepSeek excelled technically but exhibited a mechanical, less relatable tone. Claude balanced accuracy and tone but remained somewhat generic in emotional nuance. These results echo prior evaluations in which ChatGPT produced the most empathetic and guideline-consistent responses, yet often relied on formulaic expressions that, while linguistically polite, lacked contextual sensitivity (9). Conversely, studies assessing “human care” scores across medical queries found that empathy and humanistic quality were rated lower than factual accuracy, suggesting that LLMs may emulate emotional language without true emotional contextualization (9, 10). Importantly, excessive or superficial empathy may foster misplaced confidence, underscoring the need for ethically guided AI communication frameworks in patient-facing applications. Our results therefore highlight a persistent gap between medical precision and emotional authenticity—one that must be addressed if LLMs are to be safely integrated into patient education and communication.

The findings of this study suggest that large language models (LLMs) are best positioned as supportive tools for patient education and information delivery rather than as substitutes for clinical decision-making. In rare and complex diseases such as Ewing sarcoma, these systems can facilitate understanding for patients and families, helping to clarify diagnostic and therapeutic concepts. However, consistent with prior literature, the accuracy, reliability, and ethical integrity of AI-generated information remain contingent upon professional supervision, particularly in high-stakes medical scenarios such as cancer diagnosis, drug selection, and prognosis communication (11, 15). Future developments are expected to enhance LLM performance through retrieval-augmented generation (RAG) systems and domain-specific fine-tuning, which can improve factual precision and contextual relevance. Furthermore, the integration of multimodal AI systems that combine text, image, and speech-based data may enable a more comprehensive and empathetic approach to patient communication. Ultimately, the safe integration of AI into healthcare requires not only technical optimization but also robust ethical oversight and institutional accountability to ensure patient safety and trust. Despite their growing potential, LLMs remain vulnerable to hallucinations, factual inconsistency, and various forms of bias related to training data and model architecture. Such limitations may affect reliability and create challenges when AI-generated information is applied to clinical or research decision-making. In oncology and other high-stakes medical settings, inappropriate reliance on unsupervised LLM outputs may contribute to misunderstanding, inaccurate risk interpretation, or unsafe recommendations. Therefore, continued human oversight and critical validation remain essential for the responsible use of LLM-based systems.

Beyond patient education, LLMs are increasingly being explored in broader oncology-related applications including clinical decision support, literature synthesis, medical documentation, and oncology education. Nevertheless, the primary focus of the present study was patient- and family-oriented communication rather than professional-level oncologic decision-making or tumor-board support.

Several limitations of this study should be acknowledged. First, the evaluations were conducted by only two orthopedic oncology specialists; including experts from other disciplines such as medical oncology, radiology, and psychology could have provided a more comprehensive assessment. In addition, although consensus discussions were performed for discrepant ratings, the absence of a larger multidisciplinary evaluation panel may have limited the robustness of subjective assessments.

Second, only English-language responses were analyzed, precluding evaluation of multilingual performance and linguistic adaptability. Third, the dynamic nature of LLMs—where frequent model updates and version differences can lead to variability in outputs—represents an inherent methodological constraint. Despite these limitations, the study possesses notable strengths. It is, to our knowledge, the first systematic evaluation of LLM communication and accuracy in the context of Ewing sarcoma, a rare pediatric cancer. Additionally, the combined use of quantitative expert scoring and qualitative thematic analysis of empathy and clarity allowed for a multidimensional evaluation of AI-generated content. These strengths position the study as a foundational reference for future investigations into AI-assisted communication and patient education in rare and high-stakes medical contexts.

## Conclusion

In conclusion, this study provides novel evidence on the accuracy, clarity, and empathy of large language models (LLMs) in responding to disease-specific questions about Ewing sarcoma. Among the five models assessed, ChatGPT and Claude demonstrated the most consistent and clinically reliable performance, whereas DeepSeek, Gemini, and Grok exhibited variable accuracy and communication quality. These findings indicate that while current LLMs hold promise as accessible and informative tools for patient and family education, they remain limited by inconsistency, lack of contextual understanding, and superficial emotional tone. As artificial intelligence continues to evolve, its safe and effective use in healthcare will depend on structured validation, ethical governance, and integration under professional supervision. This study, therefore, contributes an important foundation for future multidisciplinary research aimed at optimizing the role of LLMs in patient-centered communication, particularly in rare and complex oncologic conditions such as Ewing sarcoma.

## Data Availability

The raw data supporting the conclusions of this article will be made available by the authors, without undue reservation.
